# Co-administration of Pregabalin and Curcumin Synergistically Decreases Pain-Like Behaviors in Acute Nociceptive Pain Murine Models

**DOI:** 10.3390/molecules25184172

**Published:** 2020-09-11

**Authors:** Sarinee Leksiri, Hasriadi, Peththa Wadu Dasuni Wasana, Opa Vajragupta, Pornchai Rojsitthisak, Pasarapa Towiwat

**Affiliations:** 1 Inter-Department Program of Pharmacology, Graduate School, Chulalongkorn University, Bangkok 10330, Thailand; sarinee.leksiri@gmail.com; 2 Pharmaceutical Sciences and Technology Program, Faculty of Pharmaceutical Sciences, Chulalongkorn University, Bangkok 10330, Thailand; adhiehasri@gmail.com (H.); dasuniwasana@ahs.ruh.ac.lk (P.W.D.W.); 3 Research Affairs, Faculty of Pharmaceutical Sciences, Chulalongkorn University, Bangkok 10330, Thailand; opa.vaj@mahidol.ac.th; 4 Department of Food and Pharmaceutical Chemistry, Faculty of Pharmaceutical Sciences, Chulalongkorn University, Bangkok 10330, Thailand; pornchai.r@chula.ac.th; 5 Natural Products for Ageing and Chronic Diseases Research Unit, Chulalongkorn University, Bangkok 10330, Thailand; 6 Department of Pharmacology and Physiology, Faculty of Pharmaceutical Sciences, Chulalongkorn University, Bangkok 10330, Thailand

**Keywords:** pregabalin, curcumin, nociceptive pain, synergistic interaction, acetic acid-induced writhing test, tail-flick test

## Abstract

Analgesic drugs in a combination-form can achieve greater efficacy with lesser side effects compared to either drug alone. The combination of drugs acting at different targets or mechanisms of action has been recognized as an alternative approach for achieving optimal analgesia. In this study, the analgesic effects of pregabalin (30, 60, 100, 200 mg/kg), curcumin (15, 30, 60, 100, 120 mg/kg), and 1:1 fixed-dose ratio of the pregabalin-curcumin combination were assessed using two acute nociceptive pain models, the acetic acid-induced writhing and tail-flick tests in mice. The pregabalin-curcumin combination produced a dose-dependent decrease in mean of writhes and an increase in the percentage of antinociception by the acetic acid-induced writhing test. In the tail-flick test, the combination also showed an improvement in antinociception indicated by the tail-flick latency, % antinociception, and area under the curve (AUC). Isobolographic analysis of interactions demonstrated a significant synergistic interaction effect between pregabalin and curcumin in both acute nociceptive pain models with the experimental ED_50_ below the predicted additive line and the combination index < 1. These findings demonstrate that the combination of pregabalin and curcumin exhibits a synergistic interaction in mouse models of acute nociceptive pain.

## 1. Introduction

Pain is a nociceptor response to noxious and non-noxious stimuli that, in turn, stimulates the central nervous system to perceive pain [1]. Physiologically, pain is a warning signal, but excessive nociceptor activation can contribute to a debilitating condition known as nociceptive pain [2]. Current pharmacotherapy for acute pain includes acetaminophen, steroids, non-steroidal anti-inflammatory drugs (NSAIDs), and opioids. However, their side effects, such as gastrointestinal bleeding, nephrotoxicity, cardiovascular events, opioid dependence, remains problematic [3]. Therefore, drug combinations are used as an alternative therapeutic strategy to enhance the efficacy and reduce the side effects of associated drugs.

One of the potent targets for pain treatment is the calcium channel [4]. The α2-δ subunits of voltage-gated calcium channels (VGCCs) are distributed and expressed in the peripheral nerves of the dorsal root ganglia and the central nervous system (CNS) [5,6]. The administration VGCC antagonists, such as gabapentin and pregabalin, resulted in behavioral analgesia in both humans and animals [7,8]. In comparison to gabapentin, pregabalin is known to be more effective and has improved pharmacokinetic properties [9]. At the mechanistic level, pregabalin binds to α2-δ subunits of VGCC and blocks the entrance of Ca^2+^ into the presynaptic neurons in the spinal cord, which reduces the release of excitatory neurotransmitters [10]. Pregabalin is considered as a first-line treatment in neuropathic pain. Moreover, pregabalin treatment reduced acute postoperative pain in humans, which might be attributed to its antinociceptive activity [11].

Nowadays, substantive consideration has been given to the use of pregabalin in the combination approach due to its potent analgesic efficacy and the ability to enhance the potency of other drugs. In acute nociceptive pain models, co-administration of pregabalin and tramadol with a weight ratio of 1:3 significantly improved nociceptive pain compared to each drug alone [12]. Pregabalin also synergistically improved pain-like behaviors when combined with acetaminophen [13]. In clinical settings, pregabalin administration combined with dexamethasone improved pain in postoperative patients with lumbar spinal surgery [14]. Despite many advantages of using pregabalin for pain treatment, its side effects remain a concern. A study in mice found that pregabalin can cause development of dependence by modulating the glutamatergic system [15]. For some instances, pregabalin causes rashes, weight gain, dry mouth, dizziness, and heart failure [16,17]. In addition, pregabalin discontinuation can also cause withdrawal symptoms [18], and the administration during pregnancy can potentially lead to a high risk of congenital disabilities [19].

Curcumin is a natural compound, abundantly found in the *Curcuma longa* rhizome, and has been used as a food ingredient for coloring and flavoring, and as a health supplement for centuries [20]. Several studies reported that curcumin produces analgesic activity by suppressing the immune response, modulating pain-associated neurotransmitters, and blocking the transient receptor potential vanilloid type I (TRPV1) receptors [21,22,23,24]. Moreover, curcuminoids (1500 mg/day) have analgesic effects in patients with osteoarthritis [25], while curcumin 500 mg/kg improves rheumatoid arthritis [26]. Concerning the safety, acute and repeated administration of curcumin has shown to be safe in animals and is categorized as “generally recognized as safe” (GRAS) for human use by the US FDA [27,28]. Despite obvious advantages, curcumin has poor water solubility, low stability, and is rapidly metabolized in the gastrointestinal tract and liver. A meager amount of curcumin reaches the systemic circulation, which reduces its oral efficacy [29]. Oral administration of curcumin in humans (2 g/kg) displays a very low amount of curcumin (0.006 ± 0.005 μg/mL) in plasma [30], while curcumin 4–8 g/kg reaches a systemic circulation of about 0.4–3.6 μM [31]. Hence, many efforts have been taken to overcome this limitation such as structural modifications, formulation modifications and combination with other drugs [32,33,34]. Considering the combination approach, curcumin has been reported to produce a synergistic interaction when combined with a cyclooxygenase inhibitor, diclofenac, in the formalin test [35]. Despite the synergic effect in the pain models, curcumin was found to have synergistic interaction with docosahexaenoic acid [36], piperine [37] in cancer and lung toxicity models, respectively. At the mechanistic level, curcumin in combination with either docosahexaenoic acid or eicosapentaenoic acid synergistically inhibited oxidative stress as well as pro-inflammatory mediators (NO, iNOS, COX2) in LPS-stimulated RAW 264.7 cells [38]. In animals, curcumin combined with sitagliptin acting synergistically reduced nephrotoxicity, oxidative stress and proinflammatory cytokines (tumor necrosis factor-α (TNF-α), interleukin 6 (IL-6) and interleukin 1β (IL-1β) in DLM (deltamethrin)-intoxicated rats [39].

The analgesic efficacy of pregabalin in nociceptive pain is frequently unsatisfactory, and the possible side effects remain a concern. Curcumin also plays a significant role in the management of both acute and chronic pain and showed potent analgesia clinically [40]. Curcumin, however, is often confronted with physicochemical and pharmacokinetic issues: low water solubility and low membrane penetration from the ionized phenolic moieties [29,41]. Hence combining other drugs with curcumin and pregabalin have been applied for the possible synergism to reduce the dose of both drugs. For example, pregabalin exhibited a synergistic effect with acetaminophen, curcumin with diclofenac [13,35]. Pregabalin and curcumin have distinct modes of action when modulating pain, making it possible to produce greater analgesics compared to their parent drug when administered as a combination. Therefore, the reason for this combination is that, in view of the fact that both mechanisms of actions may generate analgesia on their own, and they both work differently at the peripheral and central levels of pain transmission. This study evaluated the synergistic interaction of pregabalin-curcumin in acute nociceptive pain models. Here, we report the synergistic interaction effect between pregabalin and curcumin in mouse models of acute pain, the tail-flick test, and the acetic acid-induced writhing test.

## 2. Results

### 2.1. Pregabalin and Curcumin Alone Attenuate Nociceptive Visceral Pain

**Figure 1:**
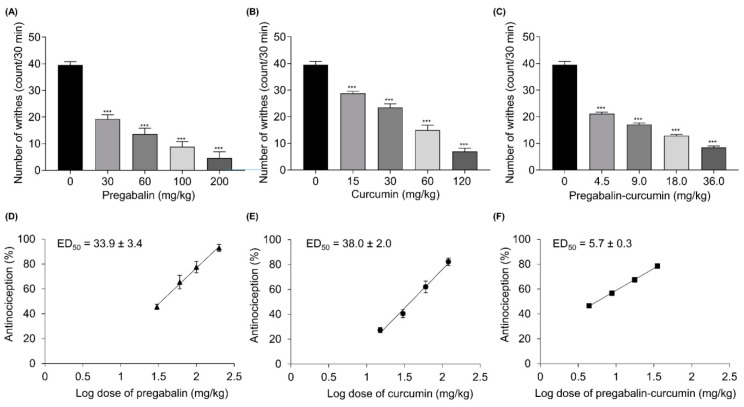
The effect of pregabalin, curcumin, and pregabalin-curcumin combination on visceral pain. The antinociceptive effect was assessed with the acetic acid-induced writhing test, presented as the mean number of writhing after the acetic acid injection (**A**–**C**) and the dose-response curves (**D**–**F**). All data represent means ± SEM. *** *p* < 0.001, versus vehicle-control group; ANOVA followed by Bonferroni.

In this study, the acetic acid-induced writhing test was conducted to assess the analgesic effects of curcumin and pregabalin on acute visceral pain. Orally administered pregabalin and curcumin individually inhibited the writhing response evoked by acetic acid, the number of writhes reduced in a dose-dependent manner (Figure 1A,B), demonstrating the anti-nociceptive activity, as illustrated by a dose-dependent increase in the percentage antinociception (% antinociception) (Figure 1D,E). Pregabalin and curcumin showed maximum % antinociception of 94.9 ± 2.6 and 82.3 ± 2.9 %, respectively. Pregabalin and curcumin treatment alone yielded the ED_50′_s at 33.9 ± 3.4 and 38.0 ± 2.0 mg/kg, respectively (Figure 1D,E)).

### 2.2. Pregabalin and Curcumin alone Attenuate Thermal Nociception

**Figure 2:**
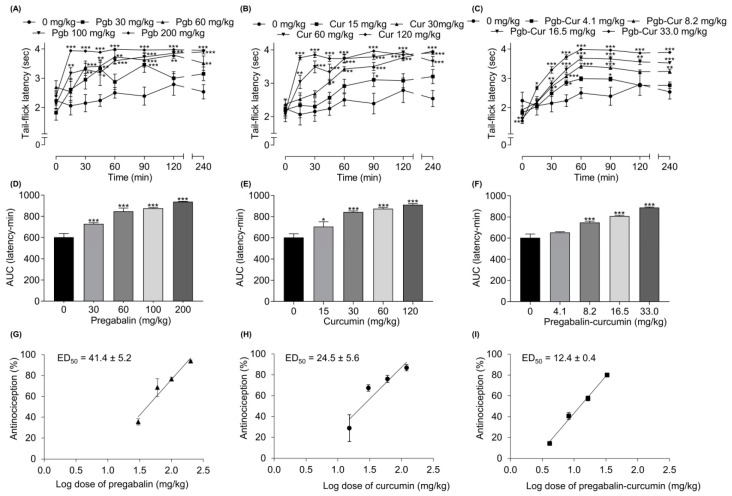
The effect of pregabalin (Pgb), curcumin (Cur), and pregabalin-curcumin (Pgb-Cur) combination on thermal nociception. The antinociceptive effect was assessed with the tail-flick test, presented as tail-flick latency (**A**–**C**), area under the curves (AUC) (**D**–**F**), and the dose-response curve (**G**–**I**). All data represent means ± SEM. * *p* < 0.05; ** *p* < 0.01; *** *p* < 0.001, versus control group; ANOVA followed by Bonferroni.

The tail-flick test was used to determine the effect of pregabalin and curcumin alone on thermal nociceptive pain. Figure 2 shows the anti-nociceptive activity of pregabalin and curcumin alone on thermal nociception with both drugs demonstrating a dose-dependent reduction in thermal nociception as represented by tail-flick latency, AUC (latency-time) and % antinociception (Figure 2) where pregabalin and curcumin yielded a maximum % antinociception of 93.9 ± 0.9 and 86.8 ± 3.0%, respectively. The analgesic action for both compounds was first observed at 15 min post-drug administration, which peaked between 60–120 min and persisted for 240 min (Figure 2A,B). The ED_50_ of pregabalin and curcumin alone were 41.4 ± 5.2 and 24.5 ± 5.6 mg/kg, respectively (Table 1).

### 2.3. Synergistic Effects between Pregabalin and Curcumin Combination on Nociceptive Visceral Pain

**Figure 3:**
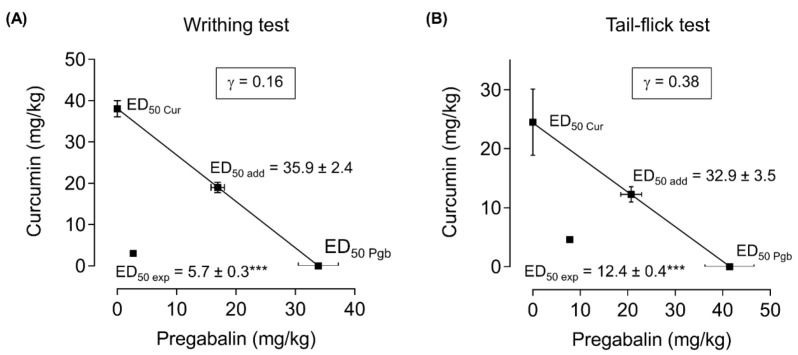
Isobologram for the antinociceptive effects of pregabalin and curcumin combination in the acetic acid-induced writhing (**A**) and tail-flick (**B**) tests. The ED_50_ for pregabalin and curcumin alone were plotted on the x-axis and y-axis, respectively. Data represented the dose (mg/kg), yielding a 50% reduction of % antinociception in both acetic acid-induced writhing and tail-flick tests. ED_50_ exp denotes experimental ED_50_ of the combination; ED_50_ add denotes theoretical ED_50_ of the combination; γ < 1 indicates a synergistic interaction; γ > 1 indicates an antagonistic interaction; γ = 1 indicates an additive interaction. γ = combination index. The experimental ED_50_ values (▪) of pregabalin and curcumin combination are below the predicted additive line, which indicates the synergistic interaction of pregabalin and curcumin. ED_50_ represented as mean ± SEM. *** represents statistical significance between ED_50_ add and ED_50_ exp analyzed by independent *t*-test (*p* < 0.001).

The pregabalin-curcumin combination (4.5, 9.0, 18.0, 36.0 mg/kg) improved nociceptive visceral pain in a dose-dependent manner illustrated by a decrease in the number of writhes and an increase of % antinociception in mice injected with acetic acid (Figure 1C,F). Further, we calculated the combination index of pregabalin-curcumin along with an isobolographic analysis to determine its synergistic interaction. Interestingly, a positive correlation exhibited between % antinociception and the total dose of fixed-dose combination, as showed in the linear regression plot (Figure 1F) and the combination ED_50_ is 5.7 ± 0.3 mg/kg (2.7 ± 0.1 mg/kg of pregabalin and 3.0 ± 0.1 mg/kg of curcumin) (Figure 1F). Isobolographic analysis also indicated the point of ED_50_ below the predicted additive line and a combination index of 0.16, indicating a synergistic interaction of pregabalin and curcumin in reducing visceral pain (Figure 3A). Moreover, theoretical ED_50_ and experimental ED_50_ values showed a significant difference (independent *t*-test, *p* < 0.05).

### 2.4. Synergistic Effects between Pregabalin and Curcumin Combination on Thermal Nociception

Orally administered pregabalin-curcumin combination (4.1, 8.2, 16.5, 33.0 mg/kg), before the application of heat with the analgesiometer in the tail-flick model revealed a dose-dependent increase in the latency with the peak effect at 60–240 min post-drug administration, as well as AUC and % antinociception (Figure 2C,F,I). The analgesic action was observed at 15 min and maintained for 240 min. To ascertain the interaction, isobolographic analysis and combination index (γ) values were calculated. The ED_50_ value for combination is 12.4 ± 0.4 mg/kg (7.8 ± 0.3 mg/kg of pregabalin and 4.6 ± 0.2 mg/kg of curcumin) (Figure 2I). Isobolographic analysis of this combination yielded an ED_50_ below the predicted additive line with the combination index of 0.38 (Figure 3B). Comparison between experimental ED_50_ and theoretical ED_50_ showed a significant difference (independent *t*-test, *p* < 0.05).

### 2.5. Co-Administration of Pregabalin-Curcumin Does not Affect Motor Coordination

**Figure 4:**
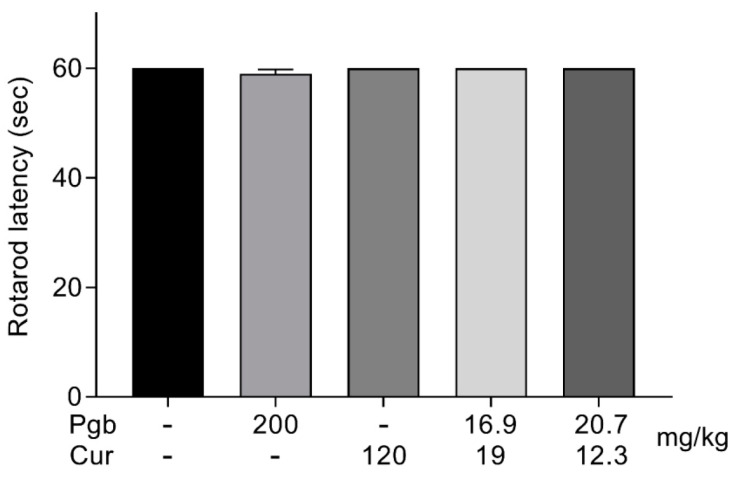
The effect of the orally administered highest doses of pregabalin (200 mg), curcumin (120 mg/kg), and pregabalin-curcumin combination (36 mg/kg in writhing test) and (33 mg/kg in tail-flick test) on motor performance by rotarod test. All data represents as latencies of the means ± SEM (n = 8/group).

All indicated doses used in this study did not display any effects on motor performances at the time points tested at 90 min post-treatment (Figure 4). These results indicate that the fixed ratio of combination at the highest doses tested did not produce any significant motor side effects to the treated mice.

## 3. Discussion

Previous reports support the potential roles of either pregabalin or curcumin to be used in the treatment of pain in a combination approach due to their ability to exert synergistic effects [13,14,35]. This study also additionally supports the synergistic interaction of co-administration of pregabalin–curcumin in attenuating acute nociceptive pain with no noticeable impact on motor function. Herein, the effects of pregabalin and curcumin alone and in combination on attenuating pain-like behaviors in acute thermal nociceptive and visceral pain models are discussed. A synergistic interaction was confirmed by the isobolographic analysis, where the ED_50_ of the combination was below the predicted additive line, and the combination index was ≤ 1.

In the present study, tail-flick and writhing tests were used to determine anti-nociceptive activity. The writhing test is initiated by administrating acetic acid, which induces an acute inflammatory response in the visceral tissues of the abdomen of mice, which then activates nociceptors [42]. Intraperitoneal injection of acetic acid induces inflammatory mediators such as tumor necrosis factor-alpha (TNF-α), interleukin 1β (IL1-β), and interleukin 8 (IL8) [43]. In addition, acetic acid also induces the release of pronociceptive agents such as aspartate, glutamate, serine, calcitonin-gene-related peptide (CGRP), and substance P [44,45]. These pronociceptive agents increase tension in the abdomen of mice causing behavioral changes, including stretching and turning, known as abdominal constriction responses [46]. In addition to the writhing test, the tail-flick test is a standard model use to assess the thermal nociceptive behavior of mice. The application of heat to the tail of mice stimulates the transient receptor potential vanilloid type I (TRPV1) and the transient receptor potential vanilloid type 3 (TRPV3) receptors, heat-sensitive receptors, thereby provoking peripheral sensitization and pain transmission [47].

In the present study, both pregabalin and curcumin alone dose-dependently decreased the pain-like behaviors in thermal nociception and visceral pain models. This study also supports the other studies where both pregabalin and curcumin showed improved analgesic effects when combined with various other compounds. Pregabalin dose-dependently produced an analgesic effect in the tail-flick, tail clip, and the acetic acid-induced writhing test, which supports the involvement of pregabalin in the opioidergic pathway. Intraperitoneal administration of pregabalin produced significant analgesia at the doses of 30–100 mg/kg and 100 mg/kg in the tail-flick test and the writhing test, respectively [48]. In another study, the most effective dose of pregabalin administered i.p. in the tail-flick test was 100 mg/kg, with analgesic effects first observed at 15 min and 60 min to peak response. The same study also found that i.p. administration of pregabalin in a ratio of 1:3 with tramadol produced a higher percentage of maximum possible effect (% MPE) compared to the individual drugs [49]. In another study, oral administration (p.o.) of pregabalin alone (20 mg/kg) produced no significant increase in the latency time but led to a significant increase in % MPE in the tail-flick test (24.5%) with the peak response at 60 min. Pregabalin alone also reduced the number of writhes in the acetic acid test, observed at 5–10 min post-drug administration. Moreover, pregabalin (20 mg/kg, p.o) significantly increased the latency time (tail-flick) and decreased the number of writhes (acetic-acid) as well as their % antinociception when combined with acetaminophen (200 mg/kg, p.o), with 15 min of the rapid onset and 30–60 min to a peak response [13]. In rats tested with tail-flick, pregabalin alone (10 mg/kg, i.p.) resulted in a 9% of %MPE whereas, in combination with μ-opioid receptor agonist, pregabalin (10 mg/kg), and oxycodone (0.6 mg/kg) elicited a 71% MPE which was significantly higher compared to each drug administered alone [50]. In combination with morphine, low dose of pregabalin (5 mg/kg pregabalin + 0.25 mg/kg morphine and 5 mg/kg pregabalin + 0.5 mg/kg morphine) exhibited higher antinociceptive effect compared to each individual drug [51]. Rats-treated with curcumin (100 mg/kg, p.o.), an onset of action, and the peak analgesic effect were observed at 60 min and 90 min, respectively in the tail-flick test [52]. In the previous study, in acetic acid-induced writhing test, 10, 20, and 40 mg/kg of intraperitoneal administration of curcumin produced antinociception of 23, 26, 29%, respectively [53]. In addition, rats treated with curcumin (20–40 mg/kg, p.o) for 8 days also demonstrated significant antinociceptive effect [54]. Considering the findings of our study, to achieve 50 percent anti-nociception (ED_50_) for visceral pain, the doses required for pregabalin and curcumin were 33.9 ± 3.4 and 38.0 ± 2.0 mg/kg, respectively, while 41.4 ± 5.2 and 24.5 ± 5.6 mg/kg, respectively, for thermal nociceptive pain. Interestingly, a lower amount of pregabalin and curcumin was used in combination to obtain ED_50_ for visceral nociceptive and thermal nociceptive pain of 5.7 ± 0.3 mg/kg (2.7 ± 0.1 mg/kg of pregabalin and 3.0 ± 0.1 mg/kg of curcumin) and 12.4 ± 0.4 mg/kg (7.8 ± 0.3 mg/kg of pregabalin and 4.6 ± 0.2 mg/kg of curcumin), respectively.

The synergistic effect of analgesic drugs usually occurs when drugs, in combination, act by multiple mechanisms at different anatomical sites [55,56]. In the present study, the synergistic interaction of pregabalin and curcumin could be due to their action on distinct mechanisms. As shown in many studies, pregabalin blocks the entrance of calcium via VGCCs, decreasing the release of spinal excitatory neurotransmitters such as glutamate, substance P, and CGRP [10,57]. In visceral pain models, the administration of the VGCC antagonist, gabapentin decreased excitatory amino acid, including serine, glutamate, and aspartate [44]. Curcumin reduces pro-inflammatory mediators (pronociceptive agents) such as cytokines, chemokine proteases, brain-derived neurotrophic factor (BDNF), cyclooxygenase-2 (COX-2), prostaglandin E2 (PGE-2), and nitrite oxide (NO) [40] Curcumin was also found to modulate neurotransmitters related to pain [22]. In a clinical study, curcumin was found to reduce serum CGRP, cytokines including tumor necrosis factor-α (TNF-α) and interleukin 6 (IL-6), and a chemokine (monocyte chemo-attractant protein 1(MCP-1)) [58,59] which are the major contributing factors in pain transmission [60]. In addition, curcumin also blocks the peripheral receptor, TRPV1, resulting in behavioral analgesia [23,24]. Altogether, pregabalin acts mainly via blocking calcium channel and curcumin via suppressing inflammatory mediators, antagonizing TRPV1, and modulating neurotransmission.

The synergistic effect of analgesics can also occur due to the actions of drugs at different anatomical sites [61]. It has been demonstrated that the main action of pregabalin is by modulating neurotransmitters in the spinal cord, though some studies also found its action probably to be in the dorsal root ganglia [10,62]. The main effect of curcumin as an anti-inflammatory agent and TRPV1 antagonist is likely in the peripheral immune system due to its inadequate blood-brain barrier (BBB) penetration. Although some studies reported that curcumin could decrease the activated resident glia in the spinal cord [63], the action might be mainly via an indirect mechanism of reducing peripheral sensitization rather than acting directly on the CNS. Therefore, the synergistic analgesia observed with the combination of pregabalin and curcumin in this study may be due to their ability to act on both the peripheral and central nervous systems concurrently. Nevertheless, the synergistic interaction of pregabalin and curcumin concerning their molecular mechanism should be interpreted with caution. Future experiments are needed to prove the synergistic interaction of pregabalin and curcumin at molecular levels.

In summary, this study demonstrates the synergistic interaction of pregabalin and curcumin in acute nociceptive pain models. Pregabalin elicited analgesic synergism on thermal nociception when combined with curcumin in both the tail-flick and acetic acid-induced writhing tests. The same therapeutic effect of individual compounds was achieved with lesser amounts of compounds present in the combination. Moreover, the experimental ED_50_ of the combination was below the predicted additive line suggesting the potential synergistic interaction. These findings support the possibility of using pregabalin and curcumin in combination for the treatment of nociceptive pain. Further studies are needed to evaluate the efficacy of this combination in other pain models, their side effects and pharmacokinetic interactions.

## 4. Materials and Methods

### 4.1. Animals

Male ICR mice weighing 18–25 g were purchased from Nomura Siam International (Bangkok, Thailand). They were housed (4–5 mice for cages) under controlled conditions: temperature (24 ± 2 °C), relative air humidity (40 to 60%) and light (12/12 h light/dark cycle), lights on at 06.00 h at the animal facility, Faculty of Pharmaceutical Sciences, Chulalongkorn University, Thailand. The animals had free access to standard laboratory food and tap water. Animals were allowed to acclimatize for a minimum of five days before the experimental procedures. All experiments were approved by the Institutional Animal Care and Use Committee of Faculty of Pharmaceutical Sciences, Chulalongkorn University, Thailand (CU-ACUP number 17-33-012).

### 4.2. Chemicals and Reagents

Pregabalin was obtained from Siam Pharmaceutical Co., Ltd. (Bangkok, Thailand). Curcumin was synthesized by the Natural Products for Ageing and Chronic Diseases Research Unit, Faculty of Pharmaceutical Sciences, Chulalongkorn University. The synthesis procedure and structural characterization results were described in Muangnoi et al. [64]. Indomethacin and carboxymethyl cellulose (CMC) were purchased from Sigma-Aldrich (St Louis, MO, USA) and acetic acid was purchased from Tokyo Chemical Industry (Tokyo, Japan). All treatments, prepared freshly prior to administration, were suspended in 0.5% CMC in saline and administered orally at 10 mL/kg.

### 4.3. Experimental Design

Animals were assigned randomly into the experimental groups (n = 8): 0.5% CMC (vehicle-control), pregabalin (30–200 mg/kg), curcumin (15–120 mg/kg) or their combinations in a fixed-ratio (1:1) of ED_50_ of each treatment alone: 1/2, 1/4, 1/8, and 1/16 × (pregabalin ED_50_ + curcumin ED_50_). The dose range of individual pregabalin and curcumin was selected based on their efficacy reported in the previous studies [48,51,53].

#### 4.3.1. Acetic Acid-Evoked Writhing Test

Acetic acid-induced writhing test was used as a representative of visceral pain in rodents [65]. Mice were randomly divided into groups of eight animals per group and allowed to acclimate for at least 30–60 min before the experiment. Visceral pain was induced by intraperitoneal injection (i.p.) of 0.6% acetic acid (w/v in saline, 10 mL/kg) [66]. The number of writhes, when the mouse showed a stretching behavior of the abdomen or extension of at least one hind limb, was determined. The writhing response was recorded at 5 min intervals for 30 min after administration. Each treatment was evaluated for analgesic effect after 60 min of a single administration.

#### 4.3.2. Tail-Flick Test

The tail-lick test was performed to evaluate the analgesic effects of compounds on acute thermal nociception [67]. A radiant light beam of the tail-flick analgesiometer (Harvard Apparatus, Holliston, MA, USA) was applied to the dorsal surface of the animal’s tail. The time from the initial application of the light beam to the withdrawal of the tail was measured and considered as the tail-flick latency. The cut-off time of 4 sec was adopted to prevent tissue damage. Mice with baseline tail-flick latency higher than the cut-off were excluded from the experiment.

### 4.4. Rotarod Test

The effects of the highest doses of individual pregabalin and curcumin and all the drug combinations on motor function were assessed using the rotarod test. The animals were placed on a horizontal rod rotating at 16 rpm. Mice capable of remaining on the rotating rod for 60 s or more in three consecutive trials were included in the study. Mice were then treated with the test drugs, and the latency to fall from the rotating road for a maximum of 5 min was recorded at 90 min after drug administration.

### 4.5. Data Analysis

#### 4.5.1. Anti-Nociceptive Effects

The results of the behavioral experiments were expressed as mean ± SEM for 8 mice per group. Graphs were constructed for each drug by plotting the number of writhing responses and tail-flick latency as a function of the doses tested. From those data, dose-response curves were plotted for each drug by plotting % antinociception of each drug against the log doses tested.

Antinociception of the drugs in the acetic acid-evoked writhing test, defined as the percentage antinociception, was quantified according to the following equation:

% antinociception = [(mean writhes in control group − mean writhes in drug(s) − treated group)/mean writhes in control group] × 100(1)

Anti-nociceptive activity in the tail-flick test, indicated by an increase of % antinociception, was calculated by the following equation [68]:

% antinociception = [(AUC_D_ − AUC_C_)/(AUC_MAX_ − AUC_C_)] ×100 (2)

AUC_D_, AUC_C_, AUC_MAX_ represent area under curve (latency × min) of treated group, control, maximum level, respectively.

#### 4.5.2. ED_50_ Calculation

The dose resulting in a 50% anti-nociceptive effect (ED_50_) was determined using linear regression according to the method previously described [69]. The experimentally derived dose-response curve for two treatment combinations was obtained by administrating curcumin and pregabalin in a constant fixed 1:1 to their average ED_50_ values in both nociceptive pain models. Finally, the experimental ED_50_ value of the pregabalin-curcumin combination was determined by linear regression.

#### 4.5.3. Isobolographic Analysis

Isobolograms were constructed using the individual ED_50_ values of pregabalin and curcumin, as described by Tallarida et al. [70]. The experimental ED_50_ value was then plotted, the effect of the combination was determined according to the location of the experimental ED_50_ value relative to the additive line.

#### 4.5.4. Calculation of the Combination Index (γ)

The theoretical ED_50_ value of the pregabalin-curcumin combination was determined, as previously described [71]. The ED_50_ values of each drug averaged across the two pain models using the equation below:

ED_50 add_ = f (ED_50 D1_) + (1 − f) (ED_50 D2_) (3)

where the ED_50add_ is the theoretical ED_50_ value for drug combination, ED_50D1_ is the average ED_50_ of pregabalin, ED_50D2_ is the average ED_50_ curcumin and f is a fraction of the fixed ratio of each drug. Then the combination index was calculated using the formula below:

γ = experimental ED_50_ (ED_50 exp_)/theoretical ED_50_ (ED_50 add_) (4)

The combination index indicates what portion of the ED_50_ of the individual drugs accounts for the corresponding ED_50_ in the combination. Hence the combination will be interpreted as additive if γ = 1, synergistic if γ < 1 and antagonistic if γ > 1.

### 4.6. Statistical Analysis

Results of all behavioral tests are demonstrated as means ± SEM. Data were analyzed by one-way analysis of variance (ANOVA) followed by Bonferonni post hoc test for multiple comparisons and *t*-test. The minimum level of statistical significance was considered to be achieved when *p* < 0.05.

## Figures and Tables

**Table 1:** Potency of pregabalin, curcumin, pregabalin-curcumin combinations in the acetic acid-induced writhing and tail-flick tests.

Compound	ED_50_^a^ ± SEM					
	Writhing Test			Tail-Flick Test		
Pregabalin	33.9 ± 3.4			41.4 ± 5.2		
Curcumin	38.0 ± 2.0			24.5 ± 5.6		
	ED_50_ ± SEM					
Combination (1:1)	ED_50 exp_^b^	ED_50 add_^c^	γ ^d^	ED_50 exp_^e^	ED_50 add_^f^	γ ^g^
Pregabalin + Curcumin	5.7 ± 0.3 ***	35.9 ± 2.4	0.16	12.4 ± 0.4 ***	32.9 ± 3.5	0.38

^a^ The dose required to produce 50% antinociception (ED_50_) ^b^ Experimental ED_50_ (ED_50 exp_) of pregabalin and curcumin combination (1:1) in writhing test. ^c^ Theoritical ED_50_ (ED_50 add_)of pregabalin and curcumin combination (1:1) in writhing test. ^d^ Combination index (γ) of pregabalin and curcumin combination (1:1) in writhing test. ^e^ Experimental ED_50_ (ED_50 exp_) of pregabalin and curcumin combination (1:1) in tail-flick test. ^f^ Theoritical ED_50_ (ED_50 add_) of pregabalin and curcumin combination (1:1) in tail-flick test. ^g^ Combination index (γ) of pregabalin and curcumin combination (1:1) in tail-flick test. *** *p* < 0.001 between ED_50 exp_ versus (ED_50 add_); independent *t*-test.

## Abbreviations

NSAIDs	Non-steroidal anti-inflammatory drugs
VGCCs	Voltage-gated calcium channels
CNS	Central nervous system
TRPV1	Transient receptor potential vanilloid 1
GARS	Generally recognized as safe
5US FDA	United State Food and Drug Administration
NO	Nitrite oxide
iNOS	Inducible nitric oxide synthase
COX-2	Cyclooxygenase-2
LPS	Lipopolysaccharide
TNF-α	Tumor necrosis factor α
IL-6	Interleukin 6
IL1-β	Interleukin 1β
IL-8	Interleukin 8
DLM	Deltamethrin
AUC	Area under the curve
ED_50_	50% Effective Dose
ED_50 add_	Theoretical ED50
ED_50 exp_	Experimental ED50
f	Fraction
γ	Combination index
	
ANOVA	Analysis of variance
Cur	Curcumin
Pgb	Pregabalin
Pgb-Cur	Pregabalin and curcumin combination
	
CGRP	Calcitonin-gene-related peptide
TRPV3	Transient receptor potential vanilloid 3
i.p.	Intraperitoneal injection
p.o.	Oral administration
% MPE	Percentage of maximum possible effect
BDNF	Brain-derived neurotrophic factor
PGE-2	Prostaglandin E2
MCP-1	Monocyte chemo-attractant protein 1
BBB	Blood-brain barrier
CMC	Carboxymethyl cellulose
